# Biosynthesis of xanthan gum from residual glycerin from biodiesel production for drilling fluids

**DOI:** 10.1186/1753-6561-8-S4-P187

**Published:** 2014-10-01

**Authors:** Lillian B Brandão, Jorge A Lopez, Denilson J Assis, Emiliano M Echevarria, Janice I Druzian

**Affiliations:** 1 Departamento de Engenharia Química, Escola Politécnica, Universidade Federal da Bahia, Salvador, BA, Brazil; 2 Programa de Pós-Graduação em Biotecnologia Industrial, Universidade Tiradentes/Instituto de Tecnologia e Pesquisa, Aracaju, SE, Brazil; 3 Laboratório Carboflex, Empresa Carboflex, Taquipe/São Sebastião do Passé, BA, Brazil; 4 Departamento de Análises Bromatológicas, Faculdade de Farmácia, Universidade Federal da Bahia, Salvador, BA, Brazil

## Background

Several reports have focused on the value and biological transformation of industrial wastes as an alternative substrate for the production of high value-added components. In this context, it can cite the crude residual glycerin obtained as the primary byproduct of biodiesel production, which has increased exponentially during the last years. As an abundant residue, its disposal into the environment entails several drawbacks and health risks [1]. The bioconversion of this by-product into added-value products by fermentation processes is an important alternative to overcoming this environmental issue. Glycerin is an attractive feedstock for the production of useful chemicals [2], since the cost of the fermentation medium represents, for example, a critical aspect of the commercial production of xanthan, which is the most important microbial polysaccharide with widespread commercial applications (*e.g*. foodstuffs, pharmaceutical, agricultural products, petroliferous industries) [3], due to its rheological properties and its capacity to produce viscous solutions at low concentrations, together with other characteristics like its pseudoplastic property. This study investigates the effect of glycerin as alternative substrate for xanthan production by *Xanthomonas campestris*, by evaluating its operational production conditions as well as their physicochemical properties, aiming at its application as drilling fluid.

## Methods

Assays were carried out in 250 mL shake flask cultures with 80 mL of medium containing 2% crude glycerin as an alternative substrate, supplemented with 0.01% urea and 0.1% KH_2_PO_4_, compared with sucrose as control under the same operational conditions. In order to obtain the xanthan, the *X. campestris mangiferaeindicae *2103 was incubated at 28ºC in a rotary shaker at 250 min^-1 ^for 120h. Samples were withdrawn at regular intervals and analyzed for concentrations of biomass, xanthan, residual substrate. The molecular weight of xanthan was estimated by size-exclusion chromatography. Its viscosity for drilling fluid was determined according to the Petrobrás N-2604 standard [4]. Rheological data were fitted to the Ostwald-de-Waele model: *μ *= *K (γ)^n-1^*, using a regression analysis to ascertain the apparent viscosity (*K, n *and *R^2^*).

## Results and conclusions

The experimental results showed that glycerin supported xanthan production with a yield of 7.23 g×L^-1^, approximately 72% higher than that obtained using sucrose as carbon source. This biopolymer exhibited a consistency index (*K*) of 6342.6 ± 0,08 mPa.s^n ^and flow rate (*n*) of 0.2068 ± 0,01, according with the parameters established by the Petrobrás N-2604 standard [4], with a minimum *K *of 1500 mPa.s^n ^and maximum *n *of 0.5. These values were 70% and 30% higher, respectively, compared to gum from sucrose fermentation. Its molecular weight varied from 28.2 to 36.2 × 10^6 ^Da, analogous value to those obtained from sucrose. Therefore, the results indicated that crude glycerin has the potential to be a cost-effective and promising alternative source of carbon for the production of non-food grade xanthan, whose rheological properties show a promising alternative use as a drilling fluid to enhance oil recovery [2,5], due to *K *and *n *satisfactory values.
